# Effect of Tai Chi Combined with Mental Imagery on Cutaneous Microcirculatory Function and Blood Pressure in a Diabetic and Elderly Population

**DOI:** 10.3390/healthcare8030342

**Published:** 2020-09-16

**Authors:** Abdulrahman Alsubiheen, Jerrold Petrofsky, Wonjong Yu, Haneul Lee

**Affiliations:** 1 Department of Physical Therapy, King Saud University, Riyadh 11451, Saudi Arabia; aalsubiheen@ksu.edu.sa; 2 School of Physical Therapy, Touro University Nevada, Henderson, NV 89014, USA; Jerrold.Petrofsky@tun.touro.edu; 3 Department of Physical Therapy, Eulji University, Seongnam 13135, Korea; 4 Department of Physical Therapy, Gachon University, Incheon 21936, Korea

**Keywords:** Tai Chi, mental imagery, diabetes, blood pressure, blood flow

## Abstract

The purpose of this study was to investigate the effects of Tai Chi (TC) training combined with mental imagery (MI) on blood pressure and cutaneous microcirculatory function in individuals with diabetes and age-matched healthy subjects. All subjects participated in a one-hour Yang style TC exercise with MI twice per week for 8 weeks. An activities-specific balance confidence (ABC) measurement, a single-leg stance (SLS), a functional reach test (FRT), systolic and diastolic blood pressure, and skin blood flow were assessed. All functional outcomes were significantly improved in both groups, and systolic and diastolic blood pressures were lower in both groups after the TC training (*p* < 0.05), but there was no significant group effect. Skin blood flow decreased in the age-matched elderly group when heat and occlusion were applied (*p* < 0.05), but no difference was found in the diabetes group. Combining TC with MI showed an improvement in functional outcomes and blood pressure but cutaneous microcirculatory function did not improve. Combining TC intervention with MI theory showed an improvement in functional outcomes and blood pressure, which showed cardiovascular benefits not only in diabetes but in age-matched healthy subjects. However, cutaneous microcirculatory function was increased only in age-matched healthy subjects.

## 1. Introduction

Diabetes is associated with numerous system impairments including loss of sensory function, especially in the extremities, reduced motor control, impaired blood flow to major organs, and impaired cardiovascular function. Impaired circulation is generally considered a major factor in the damage seen to most organ systems while glycosylation end-products can cause direct organ damage [1,2]. Diabetes can disrupt the autonomic nervous system and cause endothelial dysfunction and impairment of autonomic neurons [3,4]. This reduces functions in both the sympathetic and the parasympathetic nervous systems [5]. Lack of adequate circulation causes both damage to sympathetic neurons and their ganglia [6]. While autonomic impairment is often not seen in the patient at rest, in the presence of autonomic stressors the impairment is commonly observed in about 30% of patients with diabetes and especially when stressors are combined, such as heat exposure and orthostatic changes [7].

The vascular endothelium plays a major role in the regulation of vascular tone [8] and maintaining peripheral circulatory homeostasis [9]. Vasodilation is mediated through nitric oxide to provide increased blood supply proportional to the intensity of the exercise [8]. Endothelial dysfunction causes either reduced nitric oxide release or oxidation of released nitric oxide, making it biologically inactive. Endothelial dysfunction has been shown to be highly correlated with cardiovascular risk factors such as diabetes mellitus, high blood pressure (hypertension), and aging [10,11]. 

Tai Chi (TC) is a form of traditional exercise practiced in China to maintain optimal health. Because of the complexity of the exercise, the subject must concentrate intensely in each sequence of the intricate movements, which helps to facilitate and enhance the peripheral circulation. Diabetes and aging can affect the peripheral circulation and blood pressure [9,10,11]. In TC, subjects in a mediation state try to shift weight from side to side using slow movements and while concurrently concentrating and visualizing each sequence of the movement. This helps to enhance the circulation and the autonomic control of blood pressure [12,13]. MI is defined as “the mental representation of movement without any movement” [14]. In this process, the subject uses his memory, without actually performing any movements, to understand the sequence of the movement. Then, the subject repeats the memorized movements to actually perform the movement. This helps to use muscles efficiently and to enhance peripheral circulation [12,13]. 

Previous studies have supported the role of motor or mental imagery (MI) for relearning or re-conditioning [14,15]. Many studies have shown that TC has positive results in elderly people for improving circulation [16,17,18] and lowering blood pressure [19,20]. A previous study demonstrated that TC practitioners who practice TC for years had higher cutaneous microcirculatory function during exercise than subjects who did not practice TC exercises [16]. With sustained practice of TC, some studies have shown positive effects of TC on controlling blood pressure [20,21].

While some studies have examined MI with TC, it has not been examined in people with diabetes. The purpose of this study was to investigate the effects of TC training, combined with MI, on improving blood pressure and peripheral cutaneous microcirculatory function in people with diabetes and in an age-matched healthy subject. While TC has been investigated in this population, it has not been investigated combined with MI. 

## 2. Materials and Methods 

### 2.1. Study Population 

This study was approved by the Institutional Review Board (IRB) at Loma Linda University (LLU) (IRB approval # 5110209) and conducted at the Physical Fitness Laboratory at the School of Allied Health Professions (SAHP), Department of Physical Therapy. Subjects were recruited from the Drayson Fitness Center and the Diabetes Treatment Center at LLU. Study flyers were placed on the main bulletin board of the School of Allied Health Professions and the Drayson Fitness Center (main gym) in LLU. This flyer was also sent via email to all faculty and students in the SAHP. The Diabetes Treatment Center at LLU assisted by referring interested diabetic patients to the study research team and providing them with the study flyer if they felt they met the study criteria. 

All subjects read and signed the informed consent form before they began the study. Forty individuals with diabetes and healthy non-diabetics between the ages of 50 to 80 years old participated in this study (20 with type 2 diabetes and 20 non-diabetes counterparts). They had normal or controlled blood pressure in the range between 150/90 and 90/60. None had practiced TC in the last 4 months, and none exercised more than once per week. Subjects were excluded if they did not have normal or controlled blood pressure or a normal range of motion and muscle strength. They were excluded from the study if they: (1) took medications that could affect balance; (2) had a history of frequent falling; (3) had vision problems or orthopedic/neuromuscular/cardiovascular impairments that restricted exercise. 

### 2.2. Measurements

Subjective balance confidence when performing mobility tasks was measured using a 16-item activities-specific balance confidence (ABC) scale [22,23]. A score of zero represented no confidence at all and a score of 100 represented full confidence.

The functional reach test (FRT) was used to assess dynamic balance [24]. A one-meter-long stick was placed at shoulder level for the subjects so that they could lean forward with their dominant shoulder at 90 degrees and the extent of their reach could be assessed. The maximum reaching point of the third metacarpal was recorded. The subject was asked to repeat the measurement once they could ensure performing the test correctly. 

A single-limb stance (SLS) test was performed to assess static balance [25,26]. The subject was asked to stand on one leg about 6 inches off the floor with eyes open and arms on the hips. Time was recorded while subject was maintaining balance. 

The glycated hemoglobin A1C (HbA1C) blood test measuring the average concentration of blood glucose over a 3-month period is the most common outcome measure for glucose control. Approximately 5 µL of blood was obtained and used to measure the HbA1C level with a Food and Drug Administration (FDA) approved device, DCV Vantage Analyzer (SIEMENS^®^, Tarrytown, NY, USA). Since the HbA1C test is not affected by eating, blood samples for this test was taken without regard to when food was last eaten. 

Skin blood flow was measured using a MOOR Laser Doppler Imager (Moor LTD, Oxford, UK). The laser was in single spot mode; the spot, which was left on the skin and the reflected energy, was used to measure the skin blood flow. 

Blood pressure was measured using an Omron 7 Series electronic sphygmomanometer (OMRON Healthcare, Kyoto, Japan). The measurement was taken after rest for 15 min to avoid any confounding factors. 

### 2.3. Intervention

A certified TC instructor conducted the class for the entire program. We found that the Yang-style TC was the best to use here because it comprised important characteristics relevant to MI and to somatosensory enhancement. The symmetrical Yang Style Form consisted of choreographed techniques including; Ward-off, grasping a Sparrows Tail, Single Whip, Dragon rolls around, white crane spreads wings, Brush the knee. The physical exercise involves various types of movement including postural control, weight shifting, correct postural mal-alignment, and slow coordinated movements with synchronized deep breathing. The exercise was conducted on a thick mat and with shoes off for greater sensory enhancement and challenge. Subjects were instructed to execute and feel the movement sequence while they visually observed themselves in front of a mirror. Supervision from the instructor was provided to correct any errors in movement if needed to achieve better outcomes. Then, subjects were asked to concentrate on the sequence of each movement of the TC and memorize the exercise visually from the TC instructor before they executed any movement. The exercise was conducted over a thick mat and without shoes to allow greater sensory enhancement and challenge [27,28].

Subjects were asked to participate in 1-hour TC exercise sessions twice per week for 8 weeks. Each session consisted of 15 min of warm-up and 45 min of TC Yang-style technique teaching, and the remainder practicing the activity for the duration of the class. 

### 2.4. Procedures

Subjects were asked to rest for 15 min to achieve resting status and HbA1C blood was assessed for the diabetic group only. Then, Systolic Blood Pressure (SBP) and Diastolic Blood Pressure (DBP) were measured. Baseline measurements were taken to assess functional data using the following tools: (1) the ABC Scale, (2) the SLS test, and (3) the FRT. For skin blood flow measurements, a thermode was applied to the lower extremity to warm the skin for 6 min after the baseline skin blood flow was taken for a minute. Then, occlusion was applied using a blood pressure cuff inflated to 200 mmHg for 4 min followed by 3 min of additional blood flow recording. 

### 2.5. Sample Size Estimation

To determine the sample size, the G-power 3.1.9 software (Heinrich-Heine-University Dusseldorf, Dusseldorf, Germany) was used. To calculate the sample size, the probability of alpha error and power were set at 0.05 and 0.8, respectively with. In addition, the effect size was set at 0.25 based on Cohen [29]. Therefore, a sample size of 17 subjects per group was necessary. By estimating a drop-out rate of about 10%, 20 subjects per group needed to be recruited. 

### 2.6. Statistical Analysis

Data were analyzed using IBM SPSS Statistics for Windows, version 23.0 (IBM Corp., Armonk, NY, USA). The demographic characteristics of the subjects were compared between the diabetic and healthy subjects using the independent *t*-test for the quantitative variables and the Chi-square test for categorical variables. The normality of the outcome variables was measured using the Shapiro–Wilk Test. Since FRT was normally distributed Independent *t*-test was used to compare between groups, and a paired *t*-test was used to compare between pre and post intervention in each group. The baseline scores and differences between pre- and post-measures of ABC and SLS were compared between the two groups using the Mann–Whitney *U* test. The Wilcoxon Signed rank test was used to compare functional outcomes between pre- and post-intervention in each group. 

In each study group, comparisons between pre- and post-blood pressure measures after the TC exercise were assessed using a paired *t*-test. The changes in blood flow for the different times when exposed to heat, and in the presence or absence of occlusion, were assessed using a paired *t*-test. The level of significance was set at α = 0.05.

## 3. Results

Twenty-nine subjects completed the study (12 diabetics and 17 healthy subjects). Eleven participants did not complete the study due to conflicts with the TC class schedule. The general characteristics of the participants are shown in Table 1.

The mean ABC score improved significantly in both groups (*p* < 0.01), but no significant difference between the two groups was found (*p* = 0.17). Similar findings were observed for the FRT and the SLS test. In both groups, the mean FRT distance significantly increased after the 8 weeks of TC exercise compared to baseline (*p* < 0.01), but this difference was not significant between the two groups (*p* = 0.91). In addition, a significant increase was found in the mean SLS in both groups (*p* < 0.01), but no significant difference was observed between the two groups (*p* = 0.17). (Table 2).

**Figure 1:**
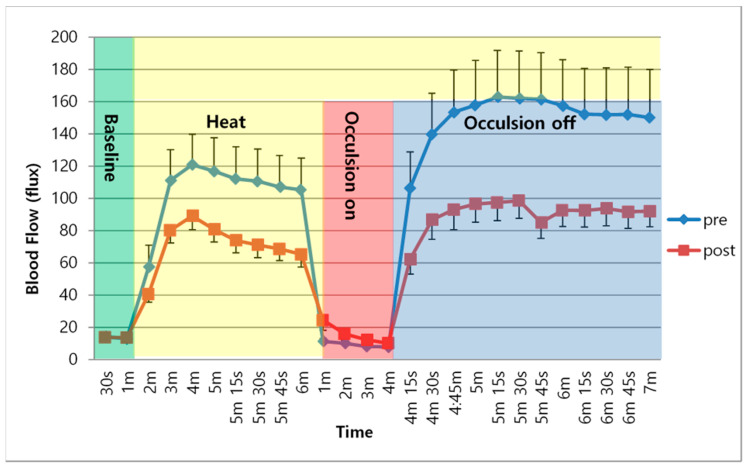
Mean and SD of Skin blood flow over time between pre- and post-intervention in healthy subjects.

**Figure 2:**
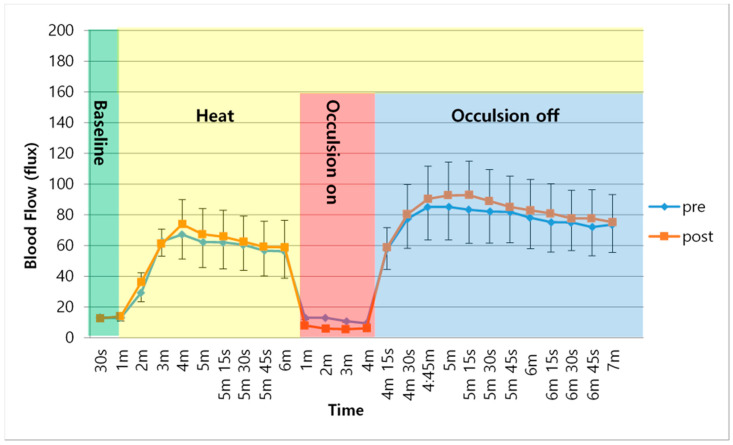
Mean and SD of Skin blood flow over time between pre- and post-intervention in diabetic group.

The determinations of skin blood flow across the two experimental conditions are shown in Table 3 and in Figure 1 and Figure 2. These figures illustrate the average skin blood flow recorded for a 6-minute exposure to heat and then after heating, and for 3 min after the occlusion of the circulation for 4 min. The response to heat is a measure of nitric oxide production by the skin since the vasodilation due to heatmediated by nitric oxide. The occlusion protocol measures vascular endothelial function. 

As shown in Figure 1, following the application of heat to the skin, skin blood flow increased for the first 3 min of exposure and then peaked, which was followed by a small reduction in skin blood flow over the subsequent 5 min in the healthy subjects. This occurred both before and after the TC training. However, the peak skin blood flow response and sustained skin blood flow after exposure to heat was significantly lower after the TC exercise in these subjects than before the TC exercise training (*p* < 0.05). After occlusion, there was even a greater reduction in skin blood flow after TC exercise. This difference was also significant (*p* < 0.05). The skin blood flow response in the diabetes group followed a similar course, as shown in Figure 2. The average resting skin blood flow, blood flow after occlusion, and increased skin temperature were significantly lower in the diabetes group than in the healthy subjects (*p* < 0.05). Furthermore, there were no significant changes in mean skin blood flow after the TC exercise when heat was on, with occlusion on, or with occlusion off (*p* > 0.05). 

The mean SBP and DBP improved significantly in both groups. The average SBP decreased significantly after the TC exercise in both diabetic patients and in healthy subjects (*p* < 0.01). However, there was no significant difference between the two groups (*p* < 0.05, Table 4). The average DBP significantly decreased after the TC exercise (*p* < 0.001, *p* = 0.03, respectively) and this difference was not significantly different between the two study groups (*p* = 0.37, Table 4). 

## 4. Discussion

In the present investigation, we assessed the effects of TC training, combined with MI, on improving blood pressure and peripheral cutaneous microcirculatory function in people with diabetes and in an age-matched control group. We found a reduction in systolic and diastolic blood pressure, and an improvement in balance as shown by an increase in motor control in both groups after TC and MI exercises. The ABC, SLS and FRT all showed significant improvements. However, skin blood flow, in response to occlusion and heat, was lower after the TC exercises in both groups. Thus there was a sensory and motor benefit to TC but the skin blood flow at rest and in response to the two stressors used here (occlusion and heat) was not improved after the TC exercise. It may be that motor control and sensory function comprise components that are independent of skin blood flow and others that are altered by blood flow. This concept is confirmed by the data reported in Table 3, which indicate motor function is increased in individuals with diabetes, although not to the same extent as in the healthy subjects. Perhaps if endothelial function had improved, the two groups would have been more similar at the end of the study, but as both groups differed, there were, therefore, other factors still able to reduce motor function in individuals with diabetes. The fact that both groups improved motor performance shows the value of Tai Chi for improving motor skills. The fact that people with diabetes showed the same improvement as healthy subjects shows that the motor control loss with ageing was more significant than any changes associated with diabetes.

Other studies have examined the effect of TC on balance. For example, a recent review showed that TC can prevent falls and improve balance in older people and in people with Parkinson’s disease [30]. However, no definitive conclusions could be drawn from this systematic review on the effect of Tai Chi on stroke, osteoarthritis and heart failure. In another Cochrane review of the effects of various types of exercise on the fear of falling in older adults, it was concluded that there was no evidence that the fear of falling was reduced after the exercise was over [31]. TC has also been shown to reduce falls in people with dementia [32,33]. A common problem in all of these studies is that the experience of the instructor and length of time TC is used and the number of times per week is variable and, therefore, it is hard to compare some of these studies to each other [34]. This was driven home in a recent systematic review of 25 studies on TC in diabetes. Here, no conclusion on the effect of TC on balance in people with diabetes could be drawn due to differences in techniques, instructors and the length of time people participated [35]. However, other reviews do agree with the present investigation in that they also found a reduction in blood pressure with TC and HbA1C [36]. In the present investigation, mental imagery during exercise was combined with TC. This has been shown to increase the H-reflex in the soleus and increase conduction velocity compared to TC alone [37]. MI seems to add a benefit to Tai Chi but as so many reviews have pointed out, comparison of studies needs more consistence in the type and duration of the exercise.

Since motor control was shown to improve in the present study equally well in age-matched healthy subjects and people with diabetes, physical therapists are encouraged to recommend TC plus MI exercise for diabetics and geriatric patients. People with diabetes, who inherently have poor balance and motor control were able to tolerate TC and complete the program. The results of this study suggest that combining a MI strategy while doing TC exercise is a potential approach to promote and accelerate the “re-learning” process, which could improve balance and gait and more importantly, prevent falls. The reduction in blood pressure seen here with TC indicates the cardiovascular benefits that may act to reduce the chance of heart disease.

However, this study has several limitations. First, 27.5% of the subjects (11 subjects) dropped out of the study. Even though the basic characteristics and baseline measurements of drop-out subjects were not significantly different from subjects who completed the study, stronger motivation should have been encouraged. Also, the small sample size due to the big drop-outs could lower the statistical power. Another important limitation of this study is the control over other daily life activities. It is unknown to what degree the TC effects achieved were derived only from TC training. Other daily physical activities or exercises may have affected the results. Another factor limiting the generalization of the results is the protective effect of anti-oxidants on endothelial function observed in different races [38,39]. Further study is needed to control for these factors to confirm an actual effect of TC with MI. 

## 5. Conclusions

In conclusion, combining TC intervention with MI theory showed an improvement in functional outcomes and blood pressure, which showed cardiovascular benefits. However, cutaneous microcirculatory function did not improve.

## Figures and Tables

**Table 1:** Demographic characteristics of participants (*N* = 29).

Characteristic	Diabetic (*n* = 12)		Healthy Subjects (*n* = 17)		*p* Value ^a^
	Mean (SD)	*n* (%)	Mean (SD)	*n* (%)	
Age (years)	63.8 (8.1)		63.6 (6.5)		0.91
Gender (Female)		8 (66.7)		13 (76.5)	0.43
Weight (Kg)	86.8 (17.2)		77.4 (17.4)		0.16
Height (m)	1.8 (0.1)		1.7 (0.1)		<0.01
BMI (Kg/m^2^)	27.9 (5.5)		27.1 (4.3)		0.66
Duration of diabetes (years)	10.8 (5.4)				
HbA1C	6.8 (0.8)				

Abbreviation: SD, standard deviations; BMI, body mass index. ^a^ *p* value from independent *t*-test.

**Table 2:** Changes in activities-specific balance confidence (ABC), functional reach test and single-limb stance test in diabetic (*n* = 12) and healthy subjects (*n* = 17).

Tool	Group		Median [Min, Max] Mean (SD)	Pre-Post *p* Value	Between Group *p* Value
ABC	Diabetic	Pre	88.8 [45.6, 95.6]	<0.01 ^a^	0.17 ^b^
		Post	95.0 [75.6, 99.7]		
	Healthy Subjects	Pre	95.0 [87.3, 100.0]	<0.01 ^a^	
		Post	96.9 [92.5, 100.0]		
Single Limb Stance	Diabetic	Pre	11.0 [2.0, 133.0]	<0.01 ^a^	0.17 ^b^
		Post	25.1 [4.7, 198.0]		
	Healthy Subjects	Pre	28.0 [4.3, 127.0]	<0.01 ^a^	
		Post	57.3 [11.3, 168.0]		
Functional Reach Test	Diabetic	Pre	11.2 (1.6)	<0.001 ^c^	0.91 ^d^
		Post	12.8 (2.2)		
	Healthy Subjects	Pre	11.7 (1.8)	<0.01 ^c^	
		Post	13.3 (1.7)		

Abbreviation: SD, standard deviation; Min, minimum; Max, maximum; ^a^ *p* value from Wilcoxon signed rank test; ^b^ *p* value from Mann–Whitney U test; ^c^ *p* value from Paired t-test. ^d^ *p* value Independent t-test.

**Table 3:** Mean (SD) of skin blood flow at pre- and post-intervention over the time in diabetes (*n* = 12) and healthy subjects (*n* = 17).

		Time	Diabetic (*n* = 12)		Healthy Subjects (*n* = 17)	
			Pre	Post	Pre	Post
**Baseline**		30 s	13.1 (2.3)	12.6 (1.8)	14.0 (1.7)	13.7 (1.3)
**Heat**	**Heat On**	1 m	13.1 (1.8)	13.9 (2.1)	12.9 (1.7)	13.5 (1.3)
		2 m	29.4 (2.5)	36.3 (5.9)	57.5 (13.5)	40.6 (5.1)
		3 m	62.7 (6.0)	61.2 (9.6)	111.2 (18.9)	80.2 (7.8)
		4 m	67.3 (7.9)	73.9 (16.0)	120.9 (18.3)	89.1 (8.5)
		5 m	62.2 (10.0)	65.8 (17.3)	116.8 (20.7)	80.7 (7.8)
		5 m 15 s	60.5 (10.2)	62.5 (16.7)	112.1 (19.9)	73.9 (7.7)
		5 m 30 s	62.5 (16.7)	56.8 (9.5)	110.6 (20.0)	71.1 (7.9)
		5 m 45 s	59.1 (16.6)	56.3 (8.9)	107.0 (19.5)	68.5 (7.2)
		6 m	56.3 (8.9)	58.9 (17.5)	105.2 (19.6)	65.2 (7.8)
	**Occlusion On**	1 m	13.0 (3.5)	8.0 (1.3)	11.3 (1.7)	24.4 (6.3)
		2 m	13.0 (3.9)	6.0 (0.6)	10.1 (1.4)	15.9 (4.0)
		3 m	10.8 (3.0)	5.6 (0.7)	8.1 (1.4)	12.1 (2.9)
		4 m	9.3 (2.4)	6.1 (0.7)	7.8 (1.2)	10.0 (2.3)
	**Occlusion off**	4 m 15 s	57.6 (10.9)	58.7 (13.1)	106.4 (22.4)	62.2 (9.2)
		4 m 30 s	77.6 (13.5)	80.5 (19.3)	139.8 (25.4)	86.7 (12.2)
		4 m 45 s	85.1 (13.9)	90.4 (21.4)	153.4 (26.1)	93.0 (12.4)
		5 m	85.3 (13.8)	92.7 (21.7)	157.8 (27.7)	96.4 (11.4)
		5 m 15 s	83.5 (13.5)	92.9 (22.1)	162.9 (28.8)	97.4 (11.2)
		5 m 30 s	82.2 (13.5)	89.0 (20.5)	162.0 (29.4)	98.5 (10.9)
		5 m 45 s	81.9 (13.5)	85.1 (20.1)	161.4 (29.0)	85.0 (9.9)
		6 m	78.2 (12.4)	82.9 (20.2)	157.4 (28.5)	92.6 (10.1)
		6 m 15 s	75.2 (12.5)	80.9 (19.4)	152.2 (28.3)	92.4 (10.4)
		6 m 30 s	75.0 (12.4)	77.7 (18.2)	151.8 (29.1)	93.7 (10.7)
		6 m 45 s	72.2 (12.0)	77.7 (18.8)	152.0 (29.4)	91.6 (10.2)
		7 m	73.6 (12.3)	75.3 (18.0)	150.1 (29.8)	91.9 (9.6)

**Table 4:** Mean (SD) of systolic and diastolic blood pressure at pre- and post-intervention over the time in diabetic (*n* = 12) and healthy subjects (*n* = 17).

Tool	Group		Mean (SD)	Pre-Post *p*-Value ^a^	Between Group *p*-Value ^b^
Systolic Blood Pressure (SBP)	Diabetic	Pre	141.4 (3.7)	0.002	0.16
		Post	128.8 (4.2)		
	Healthy Subjects	Pre	132.9 (4.9)	0.009	
		Post	120.8 (4.2)		
Diastolic Blood Pressure (DBP)	Diabetic	Pre	80.8 (2.2)	<0.001	0.37
		Post	71.6 (2.3)		
	Healthy Subjects	Pre	75.8 (2.3)	0.03	
		Post	71.0 (2.3)		

^a^*p-*value from paired *t*-test. ^b^*p-*value independent *t*-test.
